# The Effect of Nutrient Deprivation on Markers of Oxidative Stress, Inflammation, and Transcriptome in Normal and Type-2 Diabetic Human Skeletal Muscle Myoblasts

**DOI:** 10.1155/jnme/6661176

**Published:** 2025-06-10

**Authors:** Lael Ceriani, Daniel E. Newmire, Xavier F. Gonzales, Jean Sparks, Jose Guardiola, Felix O. Omoruyi

**Affiliations:** ^1^Department of Life Sciences, Texas A&M University-Corpus Christi, 6300 Ocean Drive, Corpus Christi 78412, Texas, USA; ^2^School of Health Promotion and Kinesiology, Institute for Women's Health, Texas Woman's University, 1600 N Bell Ave, Pioneer Hall, Denton 76209, Texas, USA; ^3^Department of Health Sciences, Texas A&M University-Corpus Christi, 6300 Ocean Drive, Corpus Christi 78412, Texas, USA; ^4^Department of Mathematics and Statistics, Texas A&M University-Corpus Christi, 6300 Ocean Drive, Corpus Christi 78412, Texas, USA

**Keywords:** autophagy, intermittent fasting, nutrient deprivation, nutrition, proteolysis, skeletal muscle myoblasts

## Abstract

**Background:** Intermittent fasting has become a new fad diet that may promote an environment to facilitate muscle atrophy, placing aging, and diabetic populations at risk for muscle loss due to nutrient deprivation. The purpose of this study was to investigate how nutrient availability and serum environment influence Type 2 diabetic myoblast density and viability, autophagy-related oxidative and inflammatory markers, and upstream gene expression signaling relevant to proteostasis.

**Methods:** To explore this outcome in human skeletal muscle myoblast (HSMM) and diabetic human skeletal muscle myoblast (D-HSMM), myoblast lines were cultured per standard protocol and were incubated for 12 or 24 h with either human serum (HS) or fetal bovine serum (FBS) at varying serum media concentrations: 5%, 10%, and 15%. Cell viability and density were determined; ELISAs were used to assess SOD1 and TNFα; TaqMan gene array plates were used to explore mRNA gene expression related to growth and atrophy.

**Results:** Cell viability (%) analysis showed that 0% concentration, 12 h incubation, and FBS media have lower viability (*p* ≤ 0.0001); cell density analysis showed lower values in 0% concentration and in the FBS media (*p* ≤ 0.0001); SOD1 analysis showed a scaled effect (*p* ≤ 0.05) and higher concentration in HS (12,795.07 ± 677.87 pg/mL; *p* ≤ 0.0001); TNFα concentration was higher in HSMM compared to D-HSMM (61 ± 0.82 vs. 2.52 ± 0.94 pg/mL; *p*=0.017), higher at 12 h (6.07 ± 0.88 vs. 2.50 ± 0.88 pg/mL; *p*=0.006), and higher in FBS (6.05 ± 0.88 vs. 2.08 ± 0.88 pg/mL; *p*=0.002); no meaningful increase in fold change was seen in mRNA.

**Conclusions:** Myoblast viability and density were lower in the nutrient-deprived conditions and in the FBS compared to the HS serum. The biomarker of oxidative stress was lower in the serum concentration in a scaled effect, yet higher in HS. The biomarker of inflammation was higher in the HSMM cell line, shorter incubation time period, and in FBS. HS used as a media may supply nutrients and hormonal confounders that may support or stress myoblast growth.

## 1. Introduction

Fasting, or voluntary abstinence from food and drink, has been practiced for thousands of years across various cultures. During the Paleolithic era, hunter gatherers naturally experienced cycles of feasting followed by prolonged periods without food. Many religious traditions have also promoted fasting as a spiritual and physical discipline, a practice that has persisted for millennia [1]. In recent years, intermittent fasting (IF) and time-restricted eating (TRE) have gained popularity, particularly in fitness and weight loss communities. IF involves alternating periods of eating and fasting, typically ranging from 16 to 48 h. Numerous anecdotal accounts suggest IF may aid in weight loss, lower blood pressure and cholesterol, enhance insulin sensitivity, and reduce cancer risk. Scientific studies in various model organisms, including yeasts, bacteria, nematodes, and rodents, have identified regulatory pathways that help maintain health during energy scarcity. These studies suggest a positive correlation between fasting, longevity, and disease prevention [2–4]. At the cellular level, nutrient deprivation slows protein synthesis while activating NF-κβ signaling, promoting proinflammatory cytokine synthesis [5]. Additionally, fasting triggers autophagy, a proteolytic process that degrades dysfunctional proteins and oxidizes free fatty acids to maintain metabolic homeostasis [6, 7]. This nutrient deprivation–induced autophagy is regulated by the Akt/FOXO3 axis, where FOXO3 translocation into the nucleus activates autophagy-related genes. AMPK also plays a role by promoting FOXO3 transcriptional activation, further facilitating cellular autophagy [7]. Research has demonstrated that precursor muscle cells derived from individuals with Type 2 diabetes (T2D) exhibit altered and dysregulated autophagy gene expression profiles under metabolic stress [8]. However, other studies suggest that autophagy markers in T2D skeletal muscle remain largely unaffected and adapt to hyperglycemia [9]. Given that skeletal muscle is a primary target for insulin-mediated glucose uptake, disruptions in insulin signaling in T2D may influence autophagy, potentially contributing to muscle atrophy.

Skeletal muscle loss is a common complication of T2D, particularly in aging populations [10]. While weight management is crucial for individuals with diabetes, calorie restriction poses risks, including hypoglycemic events that can lead to sarcopenia and frailty in older adults [11]. Notably, IF has been associated with a twofold increase in the risk of hypoglycemic events in individuals with T2D on fasting days [12]. Additionally, prolonged hyperglycemia in T2D contributes to muscle atrophy and reduced muscle mass [13, 14]. Although excessive nutrition can lead to insulin resistance, inadequate nutrition may also have adverse effects, as it can activate catabolic pathways and Forkhead box class O (FOXO) transcription factors, further exacerbating muscle breakdown.

Certain clinical practices advocate for prolonged fasting regimens, including water-only treatments or very low-calorie diets (under 200 kcal/day) lasting a week or more. These approaches aim to support weight management and disease prevention [1]. However, despite the reported benefits, the long-term feasibility of IF remains uncertain. Studies have found that nonobese individuals following an IF regimen experience persistent hunger, mood swings, and an increased risk of eating disorders, raising concerns about its sustainability [15, 16]. Human research on IF has yielded mixed results, highlighting the need for further investigation [15, 17–19].

The present study builds upon the thesis work of Ceriani [20], and our primary aim was to examine how nutrient deprivation and overnutrition influence myoblast density and viability, autophagy-related oxidative and inflammatory markers, and upstream gene expression signaling relevant to autophagy and muscle growth. Our secondary aim was to observe any differences found using different cell media (e.g., fetal bovine serum [FBS] compared to healthy and T2D human serum [HS]). This investigation utilizes both T2D-derived human skeletal muscle myoblasts (D-HSMMs) and nondiseased human skeletal muscle myoblasts (HSMMs). The myoblast cell model was selected due to the critical role of satellite cells in muscle repair, as they must be activated to proliferate, migrate to injury sites, and fuse with damaged fibers. Furthermore, myotube models are limited in their ability to sustain long-term cell culture experiments [21]. Additionally, it should be noted that previous research employing a nutrient starvation model on myotubes which found distinct differences in that myotube starvation does not lead to the upregulation of genes involved in the proteolytic systems, as is seen in whole muscle due to inactivity, fasting, and disease-induced muscle atrophy [22]. It was suggested that atrophy may be regulated at the protein level as opposed to the transcriptional level.

## 2. Materials and Methods

### 2.1. Skeletal Muscle Cell Line Demographics

HSMMs were collected from an individual diagnosed with T2D and a healthy person. These myoblasts were purchased and obtained from Lonza Inc., Walkersville, MD, USA (referred to as D-HSMM and HSMM, respectively). According to Lonza, the cells were isolated from donated human tissue with informed and legal consent. The D-HSMM cell line was derived from a 68-year-old Caucasian male, while the HSMM cell line was sourced from a 38-year-old Caucasian male. Further characteristics can be found in Table 1. Additionally, it is worth noting that the inherent variability in T2D phenotypes is due to genetic, lifestyle, and environmental variation [23]. Due to this variation, the metabolic responses may be more relevant to the donor and less generalizable. Our overarching goal is to explore the impact of nutrient deprivation on oxidative stress and gene expression related to growth and atrophy using a skeletal muscle cell model that may lead to further questions that will require more controlled study designs.

### 2.2. Sample and Media Preparation

Following a previously published protocol [24], HSMM (CC-2580) and D-HSMM (CC-2901) (Lonza Inc., Walkersville, MD) were cultured in Falcon flasks with skeletal muscle growth media-2 (SkGM-2 medium) containing FBS, human epidermal growth factor (HEGF), dexamethasone, L-glutamine, 30 mg/mL gentamycin, 15 μg/mL amphotericin, 50 U/mL penicillin, and 50 mg/mL streptomycin. Cells were incubated at 37°C in a humidified incubator containing 5% CO_2_. After 24 h, cells adhered to the bottom of the flask. The media was changed approximately every 48–72 h. When cells reached confluency (∼10^6^ cells/mL), cells were harvested with trypsin according to the manufacturer's instructions (Lonza Inc., Walkersville, MD).

Another focus of this experiment was to compare the traditional FBS compared to HS environments on outcome measures. A recent publication by Allen et al. suggested that traditional models of cell culture create a microenvironment that lacks physiological relevance to humans, which may lead to questioning the validity of in vitro experiments and generalizability to humans. In previous investigations, there was reported success utilizing HS or plasma microenvironments with immortalized muscle cells and human primary skeletal muscle cells, assessing proliferation, myotube diameter, and signaling related to anabolism and catabolism. Additionally, it was suggested that human-derived microenvironments may be translational and valuable to address the impact of aging, disease, and nutrient quality on skeletal muscle [25].

The experimental media was prepared by decreasing or increasing the concentration of serum in standard culture media. Lonza protocol calls for typical culture media to be 10% FBS. The experimental media either contained 15, 10, 5, or 0% serum. Other media components were not diluted and were kept at the manufacturer's recommended protocol. Different types of HS were also utilized during the experimentation. For each relative analysis, the media contained varying concentrations of either FBS (Lonza Inc., Walkersville, MD) or pooled HS samples collected from healthy or diagnosed patients with T2D (Doctors Regional Hospital, Corpus Christi, TX). The criteria used for the diagnosis of diabetes are fasting blood glucose of ≥ 126 mg/dL or a random plasma glucose sample of ≥ 200 mg/dL [26]. For the purposes of this study and the pooled serum samples collected, we categorized and compared normal HS nonfasting glucose values of ∼105 mg/dL, and diabetic human serum (DHS) nonfasting glucose as ∼200 mg/dL. Glycated hemoglobin (HbA1c) data were not available for the diabetic population.

### 2.3. Determination of Cell Concentration and Viability

Cell viability and concentration were assessed using a hemocytometer. A mixture of 100 μL of 0.4% trypan blue and cell suspension was prepared in a 1:1 ratio and vortexed in a 0.5-mL centrifuge tube, resulting in a dilution factor of 2. From this 200-μL solution, 10 μL was transferred to the hemocytometer chamber and covered with a glass coverslip. Cell counts were taken from the four corner squares and the center square of the 9 × 1-mm squares on the grid. Cell concentration was calculated using a factor of 10^4^, based on the volume of each square being 0.0001 mL (1 mm × 1 mm × 0.1 mm = 0.1 mm^3^). Viable cells appeared transparent, while nonviable cells were stained blue.

Cells were cultured using standard FBS protocol and pooled HS from both healthy individuals and those with diabetes. One hundred thousand cells were seeded and incubated in standard media for 48 h to achieve adherence and growth to approximately 10^6^ cells/mL following manufacturer-recommended procedures. After exposing HSMM and D-HSMM cells to various serum types and concentrations for 12 and 24 h, cell densities and viabilities were measured. Cell density was defined as the number of cells per mL of media, while cell viability was calculated as the percentage of live cells out of the total cell count (alive + dead) × 100.

• Cell viability was determined by the following formula:
  Percent viable = number of viable cells/number of total cells counted × 100.
• The total number of cells per unit volume was determined by the following formula:
  Cells/mL = average viable cells counted/number of squares counted × dilution factor × 10^4^.

### 2.4. Cell Plating

Initially, 10^5^ cells were seeded into each well of four 24-well polystyrene nonpyrogenic cell culture plates. The cells were incubated at 37°C with 5% CO_2_ for 48 h in SkGM-2 medium. After this incubation period, the cells adhered to the bottoms of the wells. The standard culture medium was then removed and replaced with experimental media in the designated wells. For the HS samples, HSMM cells were cultured with pooled normal serum, while D-HSMM cells were cultured with DHS.

### 2.5. Nutrient Deprivation and Excess Model

There are several nutrient deprivation models; however, to our knowledge, there are very few that have applied these conditions to a human muscle myoblast cell line derived from persons diagnosed with diabetes. A previous deprivation model was utilized in C_2_C_12_ mouse myoblast cells [27]. Additionally, it is unclear how reduced nutrient availability affects catabolic markers (atrogenes), oxidative markers, and inflammation markers in a diseased (T2D) HSMM cell line.

Both HSMM and D-HSMM cells were plated with 5%, 10%, and 15% FBS or HS. As a negative control, HSMM cells were plated with 0% serum. Additionally, a 10% positive control was used and compared to other serum concentrations. This model employed similar aspects and protocols from other nutrient deprivation experiments in other cell lines [28].

The presence or absence of dexamethasone, a synthetic steroid, was also considered in the experimental design. Dexamethasone, a glucocorticoid, is typically used to negatively regulate muscle mass. Companies such as Lonza Inc. include small amounts (0.5 mL dexamethasone/500 mL media) in standard cell culture media to prevent myoblast differentiation into myotubes and reduce inflammation [29]. Ultimately, dexamethasone was included in the experimental serum to minimize variation in media environments that may confound the data.

### 2.6. Enzyme-Linked Immunosorbent Assay (ELISA)

Sandwich ELISA were used to measure cell lysate concentrations of inflammation marker tumor necrosis factor alpha (TNFα), oxidative stress marker superoxide dismutase 1 (SOD1), and atrophy marker F-box protein 32 (atrogin-1) by the capture of and quantification of antigens found in the cell culture supernatant (Ray Biotech, Norcross, GA, and MyBioSource San Diego, CA). Prepared standards specific to the target proteins and samples were added to a 96-well plate following manufacturer-recommended procedures. The plate was then read at an absorbance of 450 nm within 30 min, following the manufacturer's protocols. The concentration of the target protein was determined from the standard curve.

Oxidative stress was assessed by measuring the concentration of SOD1 using sandwich ELISAs, comparing results to a standard curve. This determined the relative oxidative stress for each serum concentration and its relationship to muscle atrophy. Inflammation was measured by assessing the concentration of TNFα using sandwich ELISAs and comparing the results to a standard curve. This enabled us to evaluate the relative inflammation for each serum concentration and its connection to cellular stress and muscle atrophy.

### 2.7. Quantification and Purification of RNA

Cell pellets were stored in Invitrogen TRIzol reagent (Thermo Fisher Scientific) at −20°C for RNA extraction. In a small tube, 0.25 mL of the prepared mixture was combined with 0.75 mL of TRIzol, gently mixed, and incubated at room temperature for 5 min to ensure complete dissociation of the nucleoprotein complex. After incubation, 115 μL of chloroform was added, thoroughly mixed, and incubated for an additional 3 min. The samples were then centrifuged at 12,000 × g for 15 min at 4°C. This process separated the mixture into a lower red phenol–chloroform phase, an interphase, and a clear upper aqueous phase. The aqueous phase was carefully transferred to new RNase-free tubes using a pipette. RNA was then isolated through precipitation, washing, and solubilization steps. Initially, 375 μL of isopropanol was added to the tubes, and the solution was incubated at 4°C for 10 min, followed by centrifugation at 12,000 × g for 10 min at 4°C. The supernatant was discarded, and the pellet was resuspended in 750 μL of 75% ethanol. The sample was briefly vortexed and centrifuged at 7500 × g for 5 min at 4°C. After discarding the supernatant, the pellet was air-dried for 10 min and then resuspended in 50 μL of RNase-free water. RNA yield and purity were determined using a spectrometer. Absorbance at 260 nm measured total nucleic acid content, while absorbance at 280 nm assessed sample purity. RNA concentration was calculated using the formula A260/A280 × dilution × 40 = μg RNA/mL.

### 2.8. Qualitative PCR Analysis and Gene Expression

Six TaqMan customized gene expression assays (96-well fast 0.1 mL TaqMan array plates) (Thermo Fisher Scientific) were used to detect gene expression of several muscle gene markers related to growth, inflammation, atrophy, and autophagy (Table 2). These plates provided a semiqualitative assessment of gene regulation in each experimental context. While a 96-well plate was used, only 15 of the gene targets were utilized. For comparison, 18S was used as a normalization gene to express fold change. In an exploratory manner and to determine any fold change differences between serum type and concentration on myoblast mRNA expression, both Type 2 DHS and normal HS along with scaled concentrations were used in conjunction with the appropriate serum environment for each myoblast cell line (HSMM and D-HSMM). The six paired samples used were 5% HS-HSMM, 10% HS-HSMM, 15% HS-HSMM, 5% DHS-D-HSMM, 10% DHS-D-HSMM, and 15% DHS-D-HSMM. TaqMan RNA-to-Ct 1-Step Kit (Thermo-Fisher Scientific) was also used to convert RNA to Ct before using a standard cycle real-time PCR (RT-PCR) system, Quant Studio 3 (Thermo-Fisher Scientific). With a total volume of 20 μL per reaction, 0.5 μL of TaqMan RT-PCR enzyme mix (40x) was combined with 10 μL of TaqMan RT-PCR mix (2x) and 9.5 μL of RNA template + RNase-free water. The cycling conditions were aligned with the manufacturer's recommended protocol. The results for 5% HSMM were not reported in Table 3 due to the corruption of data due to incorrect cycling conditions. Results were analyzed on Thermo Fisher Cloud. The 13 genes, along with two housekeeping genes, were analyzed from HSMM and D-HSMM cells plated in different concentrations of HS after 24 h. Relative abundance was measured based on the positive control reference plate, which was the HSMM cells plated with 10% HS. The relative change in the amount of mRNA was calculated as 2^−ΔΔCt^. Results are expressed as the fold change above the normalized housekeeping genes [31].

### 2.9. Statistical Analysis

Duplicate data values were collected, and biological samples were analyzed in triplicate. Each serum concentration and time point had three biological replicates, totaling 96 biological samples. Data were obtained from either 12 or 24 h experiments. Each experiment included a negative control (0% serum) and a positive control (10% serum). PCR gene expression data were semiquantitative, with fold changes expressed in arbitrary units (A.U.). In this study, “increased mRNA expression” was defined as an N-fold ≥ 2.0, “normal expression” as an N-fold ranging from 0.5001 to 1.9999, and “decreased mRNA expression” as an N-fold ≤ 0.5 [30]. If no change in mRNA was observed, the symbol (−) was used to indicate no change. ELISA data were analyzed in duplicate and presented as the mean ± standard error of the mean (SEM). Differences among concentrations, serum, time, and cell types were evaluated using a 4-way ANOVA (4 × 2 × 2) (*p* < 0.05) with the Tukey post hoc analysis. To further isolate the main effects, both 3-way and 2-way ANOVAs were performed as necessary. Statistical analysis was completed using JMP®, v11, SAS Institute Inc., Cary, NC, 2021. Figures and graphs were created using GraphPad Prism Version 9.4.1 for Mac, GraphPad Software, San Diego, California, USA (https://www.graphpad.com).

## 3. Results

### 3.1. Skeletal Muscle Cell Viability and Density

**Figure 1:**
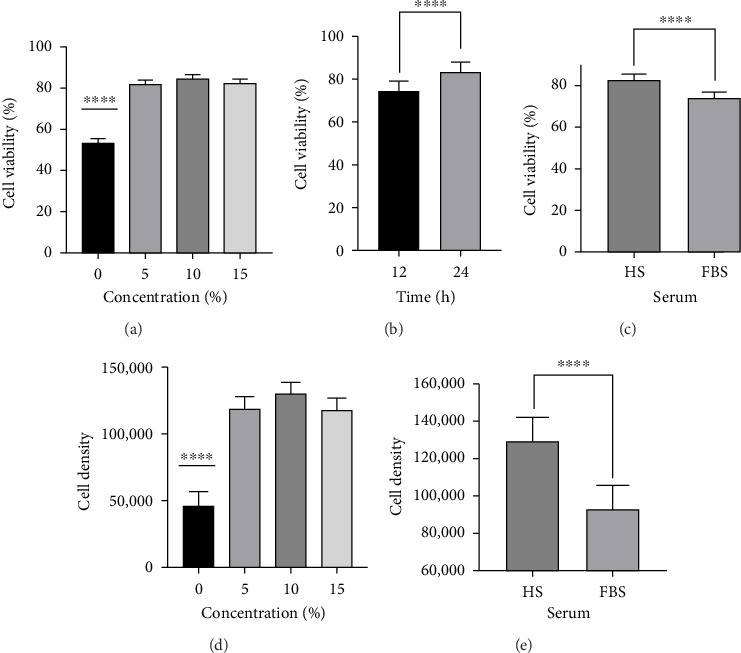
There was no effect of cell type (D-HSMM and HSMM) on cell viability (*p*=0.30). An interaction (*p* ≤ 0.0001), concentration (*p* ≤ 0.0001), time (*p* ≤ 0.0001), and serum (*p* ≤ 0.0001) effects were found. (a) 0% serum concentration had a lower (53.37 ± 2.23%; *p* ≤ 0.0001) viability value when compared to 5%–15% serum concentrations; (b) 24 h has a higher cell viability value than 12 h (83.32 ± 1.23% vs. 74.52 ± 1.22%; *p* ≤ 0.0001), (c) HS had a higher cell viability value than FBS (83.02 ± 1.22 vs. 74.5 ± 1.22; *p* ≤ 0.0001); (d) 0% concentration had a lower cell density value when compared to lower than 5%–15% concentrations (45,833 ± 11,076; *p* ≤ 0.0001); (e) HS had a higher cell density value when compared to FBS (129,672 ± 6058.08 vs. 93,610 ± 6121.43; *p* ≤ 0.0001). (^∗∗∗∗^**)** denotes a significant difference of *p* ≤ 0.0001 value.

The cell viability analysis showed that cell type had no effect (*p*=0.30) on any outcomes and was removed from subsequent analysis. Further analysis was used to assess the impact of serum concentration, time, and serum type on cell viability and density. Treatment concentrations do affect cell viability. Cells plated in 0% serum were significantly less viable, ∼53% (*p* ≤ 0.0001), than cells plated in serums with higher serum concentrations (∼82–84%) (Figure 1(a)). Time also had an effect in which the cells were more viable in the 24-h (∼83%) condition compared to 12 h (74%) (*p* ≤ 0.0001) (Figure 1(b)). The HS seemed to increase the cell viability value compared to FBS (83 vs. 74%; *p* ≤ 0.0001) (Figure 1(c)). The analysis for cell viability showed that time (*p*=0.097) and cell type (*p*=0.41) had no effect on cell density outcomes and were removed from the subsequent analysis. Further analysis showed that a serum concentration of 0% showed to have the lowest cell density value, 45,833 ± 11,076.4, compared to the other concentration values (*p* ≤ 0.0001) (Figure 1(d)). However, no differences were found between the other concentrations (5%–15%). Similar to the cell viability analysis, HS seemed to increase cell density (12,976 ± 6058) compared (*p* ≤ 0.0001) to FBS (9610 ± 6121.4) (Figure 1(e)).

### 3.2. SOD1 Concentration

**Figure 2:**
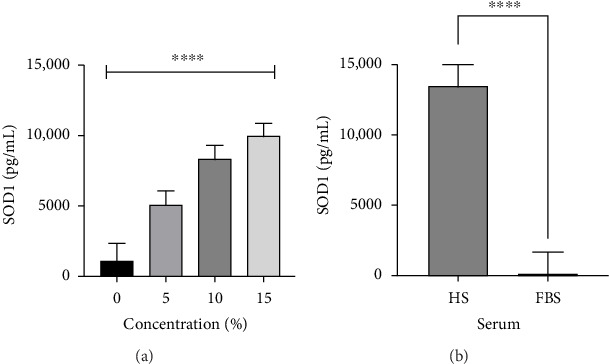
The analysis of SOD1 showed there was no effect of cell type (*p*=0.79) or time (*p*=0.87). Further analysis showed a concentration effect (*p* ≤ 0.05) and an effect of serum (*p* ≤ 0.0001). (a) Concentration of SOD1 differences were found between 0 vs. 15% (*p* ≤ 0.0001), 0 vs. 10% (*p* ≤ 0.0001), 5 vs. 15% (*p* ≤ 0.0001), 0 vs. 5% (*p*=0.04), and 5 vs. 10% (*p*=0.04). (b) A lower SOD1 concentration difference was found in FBS compared to HS (*p* ≤ 0.0001). (^∗∗∗∗^**)** denotes a significant difference of *p* ≤ 0.0001 value.

In the analysis of SOD1, there was no effect of time (*p*=0.87) or cell type (*p*=0.079), and they were removed from further analysis. Subsequent analysis showed an interaction (*p* ≤ 0.0001), an effect of concentration (*p* ≤ 0.0001), and serum type (*p* ≤ 0.0001). The post hoc analysis showed that both 0% (*p* ≤ 0.0001) and 5% (*p*=0.049) concentrations had the lowest SOD1 concentrations (1085.49 ± 1239.47 and 5124 ± 876.44 pg/mL, respectively) compared to higher 10%–15% concentrations where no difference was found (*p*=0.61) (Figure 2(a)). After the post hoc analysis, HS showed to have a higher SOD1 concentration than FBS (12,795.07 ± 677.87 vs. 245.5 ± 677.87 pg/mL) (Figure 2(b)).

### 3.3. TNFα Concentration

**Figure 3:**
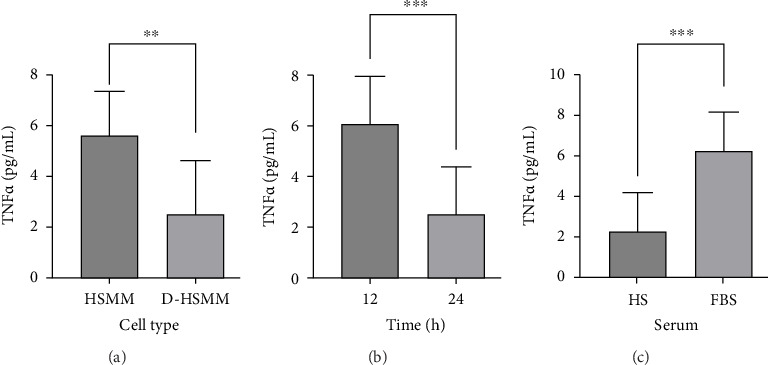
There was no effect of cell type on TNFα (*p* ≥ 0.05). (a) TNFα concentration was higher in HSMM compared to D-HSMM (*p*=0.017); (b) TNFα concentration was higher at 12 h compared to 24 h (*p*=0.006); (c) TNFα concentration was higher in FBS compared to HS (*p*=0.002). (^∗∗^) denotes a significant difference of *p*=0.01 value, and (^∗∗∗^) indicates a significant difference of *p*=0.001 value.

In the analysis of TNFα, there was no effect of serum concentration (*p* ≥ 0.05) on TNFα concentrations and was therefore removed from the subsequent analysis. Further analysis showed an interaction (*p* ≤ 0.0001), an effect of cell type (*p*=0.0082), an effect of time (*p*=0.006), and serum type (*p*=0.0025). The post hoc analysis showed a higher TNFα concentration in the HSMM cell line (5.61 ± 0.82 vs. 2.52 ± 0.94 pg/mL) compared to D-HSMM (Figure 3(a)), with higher TNFα concentration at 12 h (6.07 ± 0.88 vs. 2.50 ± 0.88 pg/mL) (Figure 3(b)), and a higher TNFα concentration found in the FBS (6.05 ± 0.88 vs. 2.08 ± 0.88 pg/mL) compared to HS (Figure 3(c)).

### 3.4. Exploratory PCR Analysis and Gene Expression

Values for gene expression in common atrophy and cell proliferation pathways were qualitatively analyzed. Table 3 depicts the fold change values in gene expression for each gene analyzed.

## 4. Discussion

### 4.1. Cell Viability/Density and Serum Concentration

Skeletal muscle is the largest protein reservoir in the human body, making protein replenishment crucial in muscle wasting. Muscle atrophy, characterized by muscle fiber loss due to metabolic diseases or toxins such as diabetes, AIDS, toxic drugs, and renal dysfunction, results from decreased protein synthesis and increased proteolysis, along with reduced regenerative capacity [32, 33]. Older Type 2 diabetics exhibit low muscle mass, likely due to both the disease and aging [10, 34]. From a myocellular perspective, myoblast fusion contributes to muscle growth and regeneration. This occurs in two phases: (1) myoblast–myoblast fusion forming initial multinucleated cells and (2) fusion of additional myoblasts with the existing myotubes, increasing myonuclei, and protein synthesis [35]. Insufficient proliferation and differentiation may lead to muscle atrophy, especially when protein degradation exceeds synthesis. The differentiation phase is regulated by specific transcription factors [36]. With additional mononuclear cells, myotube volume and protein synthesis increase, promoting myotube growth [37, 38]. A limitation of our study was the inability to confirm whether myoblasts fully differentiated into myotubes at confluency, though some spontaneous differentiation may have occurred. Additionally, it remains unclear whether the nutrient environment influenced cell proliferation or cell death, affecting observed cell density and viability. The 0% serum condition resulted in the lowest cell density, potentially simulating a muscle atrophy environment, while no significant differences were found between 5% and 15% serum, suggesting that overnutrition may not enhance muscle hypertrophy. Cell viability followed a similar pattern, decreasing significantly at 0% serum. Our findings indicate that HS more effectively promotes myoblast proliferation, as shown by increased cell density and viability compared to FBS. This aligns with the previous studies in human bone marrow–derived stromal cells, where HS was superior to FBS in supporting proliferation and differentiation potential [39, 40]. Similarly, HS was more effective in promoting human synovium–derived mesenchymal stem cell proliferation without impairing chondrogenic and osteogenic differentiation [41]. Heger et al. [42] also found that HS supported growth and migration similarly to FBS but significantly enhanced invasion and spheroid formation.

### 4.2. Oxidative Stress Biomarker SOD1

Superoxide, a harmful reactive oxygen species (ROS) primarily generated through oxygen metabolism, is typically neutralized by SOD1. This antioxidant enzyme converts superoxide into molecular oxygen and hydrogen peroxide, reducing toxicity. Our analysis found no effect of incubation time or cell line differences on SOD1 concentration. Both the HSMM and D-HSMM cell lines were plated with pooled HS, where we observed a scaled increase in SOD1 concentration in the serum across both 12- and 24-h treatment groups. We hypothesize that the increasing values from 5% to 15% serum result from exogenous SOD1 inherently present in the serum at plating. A similar trend was reported in a study comparing fluorescence emission in HS and bovine products, suggesting that differences between FBS and HS were not due to experimental effects [43]. Heger et al. [42] noted that FBS and HS differ in total protein, albumin, and estradiol concentrations, which may explain the varied effects on cell cultures observed in our study. Prior research has shown that estradiol and albumin, both found at higher concentrations in HS than in FBS, can influence mammalian cell growth and differentiation [44]. A limitation of our study was the inability to measure the presence and decay rate of enzymes already present in pooled HS.

### 4.3. Inflammation Biomarker TNFα

TNFα is traditionally recognized as a proinflammatory cytokine associated with muscle wasting and weakness in inflammatory diseases. However, some evidence suggests that TNFα may also play a role in muscle repair. A previous mammalian myocyte model demonstrated that muscle-derived TNFα acts as a mitogen, activating satellite cells into the cell cycle [45]. Our analysis found no significant impact of scaled serum concentrations on TNFα levels. However, differences were observed between cell lines, with higher TNFα concentrations in HSMM compared to D-HSMM. Given TNFα's pleiotropic effects, it is unclear whether this difference is related to myoblast catabolism or satellite cell activation. Additionally, TNFα concentration was higher at 12 h compared to 24 h, likely due to natural degradation, as TNFα has a reported half-life of ∼4.6 min and high variable circulating levels [46]. Another key finding was the notably higher TNFα concentration in FBS compared to HS, suggesting that serum composition influences TNFα levels. Previous studies using pooled HS for cell culture have reported enhanced myogenesis compared to FBS [47]. As with the cell line differences, it remains unclear whether the observed TNFα increase in serum conditions is linked to myoblast catabolism or satellite cell activation. However, given that HS promoted higher cell density and viability, this could suggest a promyogenic response, though myogenic markers were not assessed. Further investigation is needed to determine if TNFα directly influenced these outcomes. The existing research generally links TNFα to muscle catabolism through activation of the ubiquitin–proteasome pathway [48, 49]. Interestingly, our study found higher TNFα concentrations in HSMM compared to D-HSMM, contrary to our hypothesis that D-HSMM would exhibit elevated TNFα due to increased inflammatory markers associated with T2D-related muscle atrophy [50]. The reason for this discrepancy remains unclear, though the observed concentration difference was relatively small.

### 4.4. The Exploration of HS Concentrations on mRNA Expression

A semiquantitative analysis of gene expression was conducted on the 24-h HS treatment groups. HS concentrations of 5%, 10%, and 15% were used to assess the scaled effects of pooled HS on both HSMM and D-HSMM cell lines. Following our previously reported categorical approach for determining mRNA fold change, no meaningful increase (≥ 2.0) was observed in any condition. More notably, we found a relative decrease (≤ 0.5) in the D-HSMM cell line when exposed to 5%–15% HS. However, due to the exploratory nature of this analysis, it remains unclear whether this reduction was influenced by cell line, time, or serum condition. Further research is needed to clarify the impact of serum environment (HS vs. FBS), concentration, and cell line differences on the skeletal muscle transcriptome. Additionally, we observed a disconnection between TNFα mRNA and protein expression. This could be explained by the well-documented disparity between mRNA and protein concentrations [51] or by the specific posttreatment time point (24 h) selected for the analysis. Previous studies indicate that mRNA decay rates are linked to the cell cycle phase [52]. Several study limitations should be acknowledged. Age can significantly influence physiological function, and the primary cell lines used in this study were derived from donors aged 38 and 68 years. However, these were the available samples at the time of acquisition. Another limitation is that baseline serum samples collected and pooled from healthy and diabetic patients were not assessed for initial TNFα and SOD1 values, potentially confounding our analyses. Furthermore, the short observation window and limited gene expression changes in response to nutrient conditions were based on qualitative exploratory analysis rather than statistical significance. These findings serve as preliminary insights for further investigations into nutrient deficiency models on populations that may be at risk of abnormal skeletal muscle loss. The study design also did not compare all three serum types (healthy, diabetic, and FBS), restricting our ability to make direct comparisons and draw firm conclusions. Despite these limitations, this study provides valuable insights into the impact of nutrient deficiency on skeletal muscle metabolism. While dietary strategies such as IF may promote fat loss, improve insulin sensitivity, and regulate glycemia, they may also negatively affect skeletal muscle metabolism and myogenesis. Further research is needed to understand how nutrient deficiencies impact skeletal muscle, particularly in at-risk populations susceptible to muscle atrophy and loss due to caloric and nutrient restrictions.

## 5. Conclusions

Results from this study suggest that overnutrition may not confer an advantage in cell density to facilitate a greater “muscle hypertrophy” environment. The nutrient-deficient environment may promote deleterious effects on muscle cells, as demonstrated by the low cell viability and density that may promote a “muscle atrophy” environment. HS was more effective at promoting a “muscle hypertrophy” environment, as demonstrated by increased cell density compared to FBS. Our data also showed a higher concentration of TNFα in the HSMM compared to the D-HSMM. The observed difference in incubation time concerning TNFα concentration being higher at 12 h compared to 24 h may be attributable to its natural degradation. The notable increase in TNFα concentration in the FBS compared to HS, even though cell viability and density in the HS environmental condition promoted greater values, may suggest an increase in promyogenic response in the FBS environment. The gene expression analysis reflected a downregulation in several genes, primarily in the D-HSMM cell line, in all concentration conditions. However, no meaningful fold change increase was found in gene expression in either muscle cell line in any nutrient condition. Overall, implementing diets that limit nutrient availability to skeletal muscle should be performed with caution in people at risk for sarcopenia. Although the limitations of this study prevented the ability to pinpoint the optimum ranges of serum concentrations, it has revealed a need for further research in the area. Of note, when investigating the impact of nutrient and caloric factors on health-related markers, the focus should not be limited to glycemia, insulin sensitivity, fat, and adipose tissue loss, while potentially disregarding potential negative impacts that nutrient and caloric restrictions may have on skeletal muscle. The metabolic processes of human skeletal muscle are important indicators of potential whole-system effects on muscle as an organ system. Nutrient availability is a vital aspect of human health and should be especially considered among populations with diabetes and those who are aging and may be more susceptible to muscle mass loss [53, 54].

## Figures and Tables

**Table 1:** Skeletal muscle cell line donor characteristics.

	HSMM	D-HSMM
Donor age (years)	38	68
Donor race	Caucasian	Caucasian
Donor sex	Male	Male
Donor BMI	26	—
Virus testing	Not detected	Not detected
Microbial testing	Negative	Negative

**Table 2:** Genes analyzed on TaqMan array plate.

Gene name	Abbreviation	Corresponding TaqMan assay ID
Eukaryotic 18S ribosomal RNA	*18S*	*Hs99999901_s1*
Akt serine/threonine kinase 1	*AKT1*	*Hs00178289_m1*
Akt serine/threonine kinase 2	*AKT2*	*Hs01086102_m1*
Calpain 2	*CAPN2*	*Hs00965092_m1*
F-box protein 32 (atrogin-1)	*FBXO32*	*Hs01041408_m1*
Forkhead box O1	*FOXO1*	*Hs01054576_m1*
Forkhead box O3	*FOXO3*	*Hs00818121_m1*
Insulin growth factor 1	*IGF1*	*Hs01547656_m1*
Interleukin-1 beta	*IL1B*	*Hs01555410_m1*
Interleukin-6	*IL6*	*Hs00985639_m1*
Myostatin	*MSTN*	*Hs00976237_m1*
Protein kinase AMP-activated catalytic subunit alpha 1	*PRKAA1*	*Hs01562308_m1*
Ribosomal protein S6 kinase B1	*RPS6KB1*	*Hs00177357_m1*
Tumor necrosis factor alpha	*TNFa*	*Hs00174128_m1*

**Table 3:** Myoblast mRNA fold change.

Gene	HSMM		D-HSMM		
	10% HS	15% HS	5% DHS	10% DHS	15% DHS
*18S*	—	—	—	—	—
*AKT1*	1	*0.724*	**0.04**	**0.027**	**0.064**
*AKT2*	1	*1.378*	**0.031**	**0.024**	**0.064**
*CAPN2*	1	*0.865*	**0.031**	**0.026**	**0.06**
*FBXO32*	1	**0.125**	**0.081**	**0.092**	**0.206**
*FOXO1*	1	**0.369**	**0.049**	**0.033**	**0.133**
*FOXO3*	—	—	—	—	—
*IGF1*	—	—	—	—	—
*IL6*	1	—	**0.011**	**0.01**	**0.027**
*IL1B*	—	—	—	—	—
*MSTN*	1	*1.187*	**0.049**	**0.093**	**0.232**
*PRKAA1*	1	*0.563*	**0.022**	**0.033**	**0.062**
*RPS6KB1*	1	—	**0.016**	**0.028**	**0.081**
*TNFa*	—	—	—	—	—

*Note:* Normal mRNA expression (italics) N-fold ranging from 0.5001–1.9999; decreased (bold) in mRNA expression N-fold ≤ 0.5; no change (—) is defined as no increase or decrease in mRNA expression [30].

## Data Availability

The datasets analyzed during the current study are available from the corresponding author upon reasonable request.
